# Uncovering Novel Genomic Regions and Candidate Genes for Senescence-Related Traits by Genome-Wide Association Studies in Upland Cotton (*Gossypium hirsutum* L.)

**DOI:** 10.3389/fpls.2021.809522

**Published:** 2022-01-05

**Authors:** Qibao Liu, Libei Li, Zhen Feng, Shuxun Yu

**Affiliations:** ^1^State Key Laboratory of Cotton Biology, Institute of Cotton Research, Chinese Academy of Agricultural Sciences, Anyang, China; ^2^College of Advanced Agricultural Sciences, Zhejiang A&F University, Hangzhou, China

**Keywords:** cotton, senescence, GWAS, genomic region, candidate gene

## Abstract

Senescence in plants is a complex trait, which is controlled by both genetic and environmental factors and can affect the yield and quality of cotton. However, the genetic basis of cotton senescence remains relatively unknown. In this study, we reported genome-wide association studies (GWAS) based on 185 accessions of upland cotton and 26,999 high-quality single-nucleotide polymorphisms (SNPs) to reveal the genetic basis of cotton senescence. To determine cotton senescence, we evaluated eight traits/indices. Our results revealed a high positive correlation (*r*>0.5) among SPAD value 20 days after topping (SPAD20d), relative difference of SPAD (RSPAD), nodes above white flower on topping day (NAWF0d), nodes above white flower 7 days after topping (NAWF7d), and number of open bolls on the upper four branches (NB), and genetic analysis revealed that all traits had medium or high heritability ranging from 0.53 to 0.86. Based on a multi-locus method (FASTmrMLM), a total of 63 stable and significant quantitative trait nucleotides (QTNs) were detected, which represented 50 genomic regions (GWAS risk loci) associated with cotton senescence. We observed three reliable loci located on chromosomes A02 (A02_105891088_107196428), D03 (D03_37952328_38393621) and D13 (D13_59408561_60730103) because of their high repeatability. One candidate gene (Ghir_D03G011060) was found in the locus D03_37952328_38393621, and its *Arabidopsis thaliana* homologous gene (AT5G23040) encodes a cell growth defect factor-like protein (CDF1), which might be involved in chlorophyll synthesis and cell death. Moreover, qRT-PCR showed that the transcript level of Ghir_D03G011060 was down-regulated in old cotton leaves, and virus-induced gene silencing (VIGS) indicated that silencing of Ghir_D03G011060 resulted in leaf chlorosis and promoted leaf senescence. In addition, two candidate genes (Ghir_A02G017660 and Ghir_D13G021720) were identified in loci A02_105891088_107196428 and D13_59408561_60730103, respectively. These results provide new insights into the genetic basis of cotton senescence and will serve as an important reference for the development and implementation of strategies to prevent premature senescence in cotton breeding programs.

## 1. Introduction

Cotton (*Gossypium* spp.) is an important renewable natural fiber crop and an oilseed plant. As a cash crop, cotton makes a significant contribution to revenue and is thus referred to as “white gold” in several countries (Ali et al., 2014). Although improving the yield and quality of cotton is a major breeding objective, the effect of senescence also deserves attention. Plant senescence is the last stage of mature cell development, which aims to degrade cellular components and reuse them (Thomas et al., 2003; Jansson and Thomas, 2008). This stage is first dependent on the age and developmental progress, but is also regulated by diverse environmental factors such as temperature, darkness, pathogen infection, and nutrient deficiencies (Guo and Gan, 2012). In this regard, senescence is important for plants to adapt to different environments and survive under stress (Woo et al., 2019).

Although senescence is an indispensable stage of plant development, it may lead to a reduction in crop yield, particularly premature senescence induced by stress conditions. The process of senescence significantly influences the remobilization of nutrients from the vegetative plant parts to the reproductive tissues. In addition, a positive correlation between crop plant productivity and senescence is valid. For example, it has been reported that stay-green increases the yield of maize, wheat, sorghum, and rice (Jordan et al., 2012; Christopher et al., 2018; Liu et al., 2020; Shin et al., 2020). As a natural fiber crop, cotton production is also affected by senescence. Interestingly, the premature senescence of cotton is usually associated with early maturity. Although early maturity is an important goal of cotton breeding, premature senescence is a phenomenon that should be avoided in cotton breeding and production. Compared to cotton plants with normal senescence, those at premature senescence can be identified by several unique morphological features, including early wilting of the upper leaves, small bolls and poor upper boll setting (Chen and Dong, 2016). Premature senescence in cotton plants can lead to a reduction in the final lint yield and fiber quality, including fiber length, fiber strength and maturity (Wright, 1999; Dong et al., 2006). In addition, various physiological and molecular parameters change during plant senescence. For example, leaf yellowing occurs by chlorophyll degradation, which is the most visible symptom (Diaz et al., 2006). During the loss of green color, chlorophyll undergoes complex changes from its colored derivatives to colorless products (Archetti et al., 2009). Together with the strong positive correlation between the degradation of chlorophyll and photosynthesis decrease, the chlorophyll content is the most popular trait for quantifying leaf senescence (Jiang et al., 2004; Ougham et al., 2008; Bresson et al., 2017; Zhao et al., 2019).

Many senescence-associated genes (SAGs) have been identified by analysis of mutants altered in senescence phenotype. Several genes, such as *NONYELLOW- INGs/STAYGREENs* (*NYEs/SGRs*), have been identified as chlorophyll catabolic genes (CCGs) (Woo et al., 2019), which affect the breakdown of chlorophyll. Furthermore, transcription factors (TFs), such as NAC and WRKY families, also play an important role in the regulation of SAGs expression in leaf senescence (Kim et al., 2018). Moreover, the genetic basis of senescence in mapping populations and natural populations has been revealed in *Arabidopsis thaliana*, rice, soybean, maize and wheat (Hebbar et al., 2005; Shi et al., 2019; Zhao et al., 2019; Wang et al., 2020; Zhang et al., 2020a). Recently, a QTL mapping study uncovered the lifespan and senescence patterns in rice using a cross between the *Indica* and *japonica* cultivars (Shin et al., 2020). The authors suggested that promoter variations in the *OsSGR* gene could accelerate senescence in indica-type rice. In cotton, transcriptome and proteomic analyses of leaf senescence have demonstrated that leaf senescence programs are highly complex and regulated by multiple layers (Lin et al., 2015; Zheng et al., 2017). NAC genes (*GhNAC12/18*) and WRKY genes (*GhWRKY27/42/91*) have been identified as key regulators in cotton senescence (Ondati et al., 2016; Zhao et al., 2016; Gu et al., 2018, 2019a,b). However, the research on the genetic basis of natural variation in cotton senescence remains limited.

In this study, we evaluated eight senescence-related traits in 185 cotton accessions in multiple environments, and then performed multi-locus GWAS using SLAF-seq data. Our main objectives were to (i) assess the degree of variation in senescence-related traits among cotton genotypes, (ii) identify the genomic risk loci associated with cotton senescence in a diverse panel of upland cotton, and (iii) determine the candidate genes controlling the cotton senescence process.

## 2. Materials and Methods

### 2.1. Plant Materials

A cotton association mapping panel of 185 diverse accessions was selected for this analysis, as previously reported (Supplementary Table S1) (Su et al., 2016). These accessions were maintained at the gene bank of the Institute of Cotton Research of Chinese Academy of Agricultural Sciences (ICR-CAAS). According to cotton growing regions and agroclimatic zones, the germplasm originated from the Yellow River Region (YRR), the Yangzi River Region (YZRR), the Northwest Inland Region (NIR), the Northern Specific Early-Maturity Region (NSER), and foreign country (the USA). During 2013 and 2014, all of the cotton accessions were planted in two different sowing seasons: spring (designated as C2013 and C2014) and summer (designated as X2013 and X2014), at Anyang, Henan (36°08′*N*, 114°48′*E*). A completely randomized block experimental design was used in the field experiment with three replicates. Each accession was grown in single-row plots of 4 m length with a spacing of 20 cm between plants and 30 cm between rows.

### 2.2. Phenotyping and Data Analysis of Senescence-Related Traits

To determine the cotton senescence, agronomic traits were evaluated including the chlorophyll content at three time points, the relative SPAD difference (RSPAD), nodes above white flower (NAWF) at two time points, the number of open bolls on the upper four branches (NB) and the relative difference of boll weight (RDBW). The detailed investigation method followed in this study is described below, and a sketch map is provided in Supplementary Figure S1.

The chlorophyll content of the third top leaf from five plants in the middle of each row was measured after topping, using a chlorophyll meter SPAD-502 (Konica Minolta, Japan). The SPAD readings were recorded at 0, 10, and 20 days after topping (designated as SPAD0d, SPAD10d, and SPAD20d, respectively) in each environment. The RSPAD was calculated under the formula of RSPAD (%) = (SPAD20d – SPAD0d) / SPAD20d ×100%.

Although NAWF is an indicator of crop growth and development, it can be used as an indicator of cotton maturation (Bourland et al., 1992; Thompson et al., 2019). We counted the NAWF of each row on the day of topping and 7 days later (designated as NAWF0d and NAWF7d) in 2013 (during the spring sowing season) and 2014 (both sowing seasons).

Because premature senescence severely affects the maturity of cotton bolls, we counted NB before harvest in 2013 (spring sowing only) and 2014 (both sowing seasons). Ten plants were measured for each accession in each replicate. Furthermore, RDBW was calculated using the formulas RDBW (%) = (UBW – MBW) / UBW × 100, where the upper and middle bolls from 10 plants of each accession were harvested and weighed to reckon the upper boll weight (UBW) and the middle boll weight (MBW) in 2014 (spring and summer sowing).

The BLUP values and broad-sense heritability (*H*^2^) for these traits were calculated using the R package sommer (Giovanny, 2016). Broad-sense heritability was defined as $H^{2}=\sigma_{g}^{2}/\left(\sigma_{g}^{2}+\sigma_{gl}^{2}/l+\sigma_{gy}^{2}/y+\sigma_{e}^{2}/rly\right)$, where $\sigma_{g}^{2}$ is the genotypic variance, $\sigma_{gl}^{2}$ is the interactions of genotype with location, $\sigma_{gy}^{2}$ is the interactions of genotype with year, $\sigma_{e}^{2}$ is the error variance; l, y and r are the number of locations, years and replications. Statistical and correlation analyses were performed using the R package Hmisc (Harrell and Dupont et al., 2021), and then visualized with a corrplot (Wei and Simko, 2021).

### 2.3. SNP Genotyping

Detailed information on DNA extraction and SLAF-seq data has been reported in a previous study (Su et al., 2018). The quality of paired-end reads from 185 accessions was evaluated with FastQC v.0.11.9 (Andrews, 2010), and controlled using Trimmomatic v.0.39 (Bolger et al., 2014). All high-quality clean reads were mapped to the *Gossypium hirsutum* cv. TM-1 reference genome (Wang et al., 2019) with BWA mem v.0.7.17 (Li, 2013). The mapping results were sorted and converted to the bam format using Picard tools (http://broadinstitute.github.io/picard). GATK v.4.1.8 (Van der Auwera and O'Connor, 2020) was used to detect variants following the best-practice workflows. High-quality SNPs were filtered with: “QD <2.0 QUAL <30.0 FS > 60.0 MQ <40.0 MQRankSum < −12.5 ReadPosRankSum < −8.0”, missing rate <50% and MAF > 0.05.

### 2.4. Multi-Locus Genome-Wide Association Analysis

A multi-locus model was used in this study by a fast mrMLM algorithm (FASTmrMLM) in the mrMLM package (Wen et al., 2018; Zhang et al., 2020b). Before carrying out GWAS, the SNPs were imputed with Beagle v.5.1 (Browning et al., 2018). Both the BLUP and phenotypic data of each single environment were used to perform GWAS. To correct the effect of population structure, the principal components were calculated using Eigensoft v8.0.0 (Price et al., 2006). The significant and stable QTNs were selected with the following criteria: LOD values of signals must be >3.0 for at least one BLUP or two single-environment phenotypic data. The GWAS risk loci were characterized by pairwise *r*^2^>0.1 of QTNs, which were calculated by PLINK (Purcell et al., 2007).

### 2.5. RNA Isolation and qRT-PCR

For RNA isolation, cotton plants were planted in a greenhouse, and 2-week-old (young) and 8-week-old (old) leaves were sampled from ten individuals with three biological replicates in each group. Total RNA was extracted using an RNA Purification Kit (Tiangen), and the RNA was reverse transcribed using the PrimeScript RT Reagent Kit (TAKARA) following the manufacturer's instructions. Quantitative RT-PCR was performed on a Roche Applied Science LightCycler 480 (Roche) using NovoStart® SYBR qPCR SuperMix Plus (Novoprotein). The primer pairs used for qPCR analysis are listed in Supplementary Table S7. The relative expression of genes was calculated via the 2^−Δ*ΔCT*^ method (Livak and Schmittgen, 2001).

### 2.6. Prediction of Candidate Genes

All genes located in the GWAS loci were selected as putative candidate genes. The homologs of these genes in *Arabidopsis thaliana* were determined using BLAST (Camacho et al., 2009), and the functional annotation information, including the GO and KEGG terms, was obtained from the cottongen database (Yu et al., 2014). The expression patterns of genes were determined using RNA-seq data obtained from the NCBI SRA database (PRJNA532694), which were collected from *G. hirsutum* acc. TM-1 at 0, 6, 12, 24, 48 and 72 h after stress treatments, including salt, PEG and *Verticillium wilt* (VW). Differentially expression genes were analyzed using the R package edgeR (Robinson et al., 2010).

### 2.7. Virus-Induced Gene Silence in Cotton

For the VIGS assays, one fragment of *GhCDF1* (Ghir_D03G011060) amplified from the cDNA of “CRI50” was integrated into the pTRV2 vector (pTRV2-GhCDF1) using the Nimble Cloning method (Yan et al., 2020), and then the recombinant vector was introduced into *Agrobacterium tumefaciens* GV3101. Agrobacterium strains harboring the pTRV2-GhCDF1 and pTRV2 (negative control) vectors combined with strains harboring the pTRV1 vector were co-transferred into the cotyledons of 2-week-old cotton plants following previously described methods (Gao et al., 2011). And the pTRV2-PDS was used as the indicator. When the leaves of the pTRV2-PDS plants displayed an albino phenotype, leaves from the injected plants were collected for qRT-PCR analysis. Moreover, SPAD values were also determined. The primers used for the construction of the VIGS vector and qRT-PCR are listed in Supplementary Table S7.

## 3. Results

### 3.1. Phenotype Statistics

**Figure 1:**
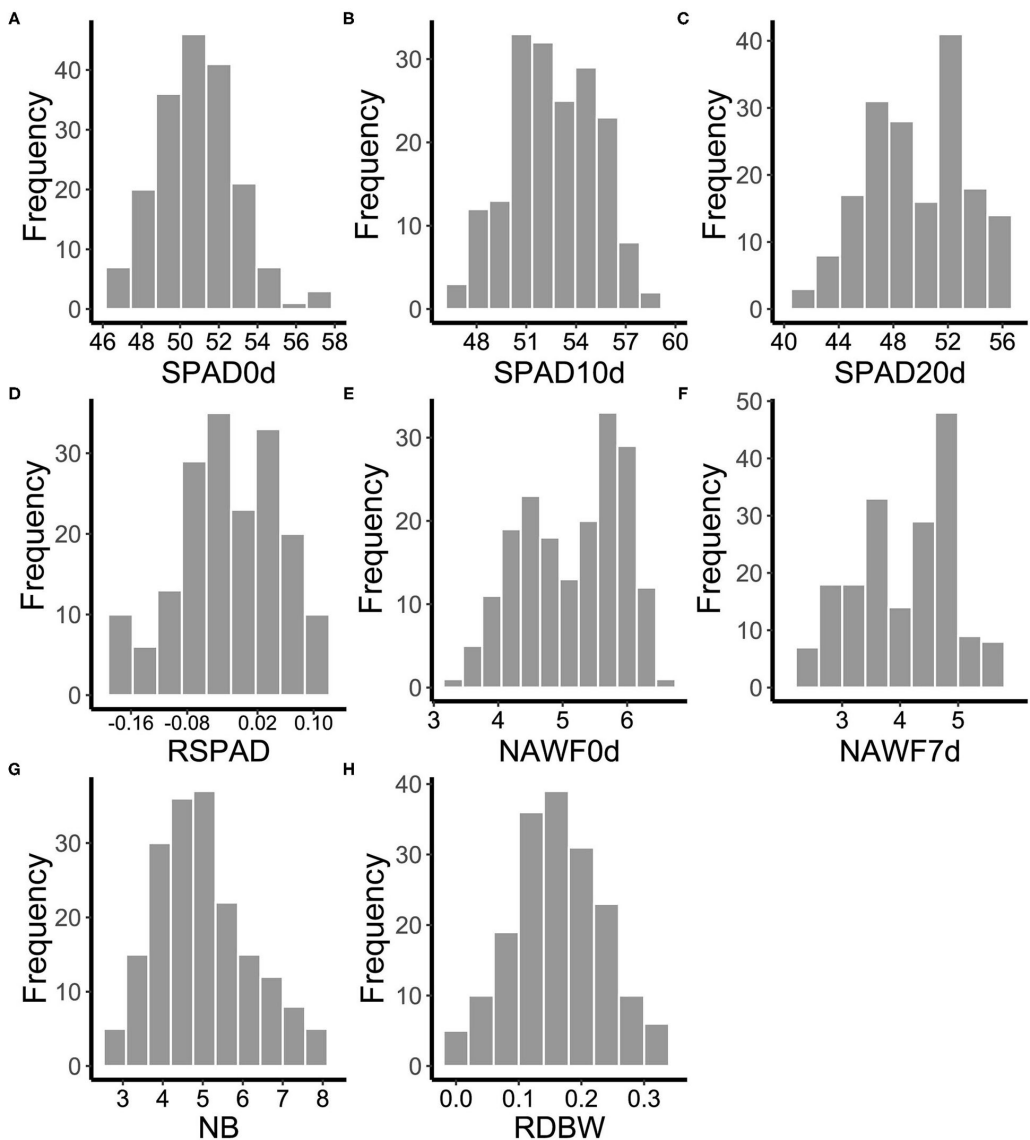
Frequency distributions of the mean values of eight senescence-related traits for 185 cotton accessions in multi-environments. **(A)** SPAD0d: SPAD value on topping day. **(B)** SPAD10d: SPAD value 10 days after topping. **(C)** SPAD20d: SPAD value 20 days after topping. **(D)** RSPAD: relative difference of SPAD. **(E)** NAWF0d: nodes above white flower on topping day. **(F)** NAWF7d: nodes above white flower 7 days after topping. **(G)** NB, number of open bolls on the upper four branches. **(H)** RDBW, relative difference of boll weight.

One hundred and eighty-five cotton genotypes were planted in multiple environments to evaluate the range of phenotypic variation of eight senescence-related traits (SPAD0d, SPAD10d, SPAD20d, RSPAD, NAWF0d, NAWF7d, NB, and RDBW). The mean performance and the frequency distribution of these traits varied with different growth conditions (Supplementary Figure S2), suggesting that senescence is significantly affected by external environmental factors. However, as expected, the mean values of SPAD first increased and then decreased during the leaf development stages, measuring 50.82 on the topping day, reaching the highest level (52.46) on the 10th day after topping, and falling to 49.49 on the 20th day after topping (Figures 1A–C and Supplementary Table S2). Moreover, the mean of NAWF value on the 7th day after topping was lower than that on the topping day (5.16 and 4.06, respectively) (Figures 1E,F and Supplementary Table S2). These results showed that SPAD and NAWF were dynamic indices of cotton growth and could be used to indicate the cotton senescence. In addition, other traits (RSPAD, NB, and RDBW) also showed wide variation in upland cotton accessions (Figures 1D,G,H and Supplementary Table S2).

**Figure 2:**
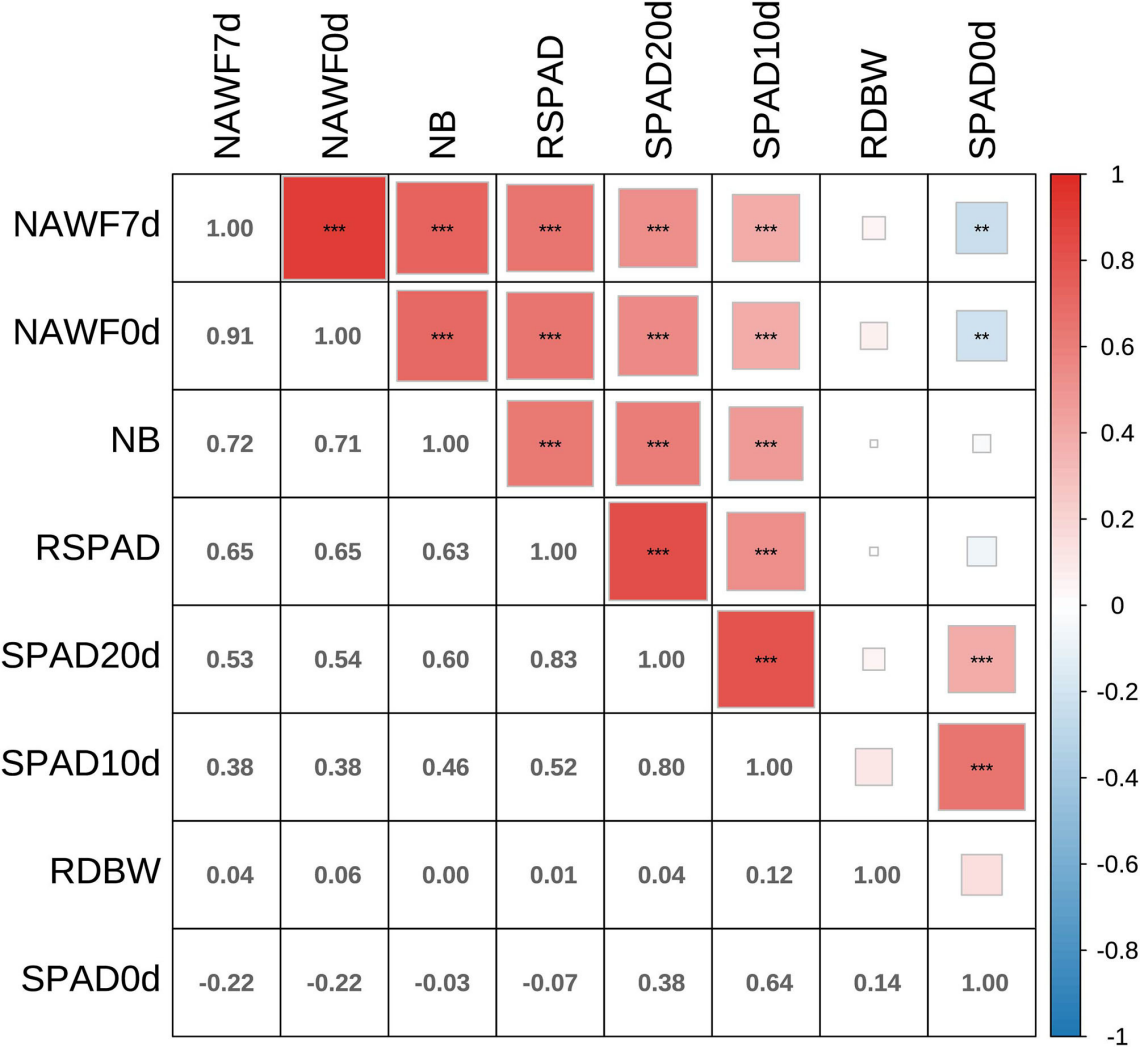
Correlation analyses between eight senescence-related traits. Asterisks indicate significance levels (***P <0.001, **P <0.01, and *P < 0.05).

The Pearson's product moment correlation coefficients and test statistics were evaluated for senescence traits, and most of them showed moderate to high correlations (Figure 2). In particular, significantly positive correlations (P < 0.001) were observed among the SPAD10d, SPAD20d, RSPAD, NAWF0d, NAWF7d, and NB indices. In addition, medium or high broad-sense heritability (*H*^2^) was found for each traits, and ranged from 0.53 to 0.86 (Supplementary Table S2). NAWF had relatively high heritability, with values of 0.86 ± 0.05 and 0.85 ± 0.05 for NAWF0d and NAWF7d, respectively.

### 3.2. Multi-Locus GWAS and Genomic Regions for Senescence Related Traits

A total of 26,699 high-quality SNPs were revealed after strict SNP calling and filtering pipelines. We then performed a genome-wide association study (GWAS) with a fast multi-locus random-SNP-effect mixed linear model (FASTmrMLM) for both single traits across different environments and for BLUP across all environments (Supplementary Figures S3–S6). Based on this analysis, 224 significant QTNs (*LOD*>3.0) were identified for eight senescence related traits (Supplementary Table S3). To reduce the number of false positives, we retained only the QTNs associated with BLUP or found in at least two environments, and 63 significant and stable QTNs were obtained (Supplementary Table S4). To determine the genomic regions (GWAS risk loci) associated with senescence, QTNs were merged if the pairwise *r*^2^>0.1. Finally, 50 genomic loci related to cotton senescence were identified (Supplementary Table S5).

**Figure 3:**
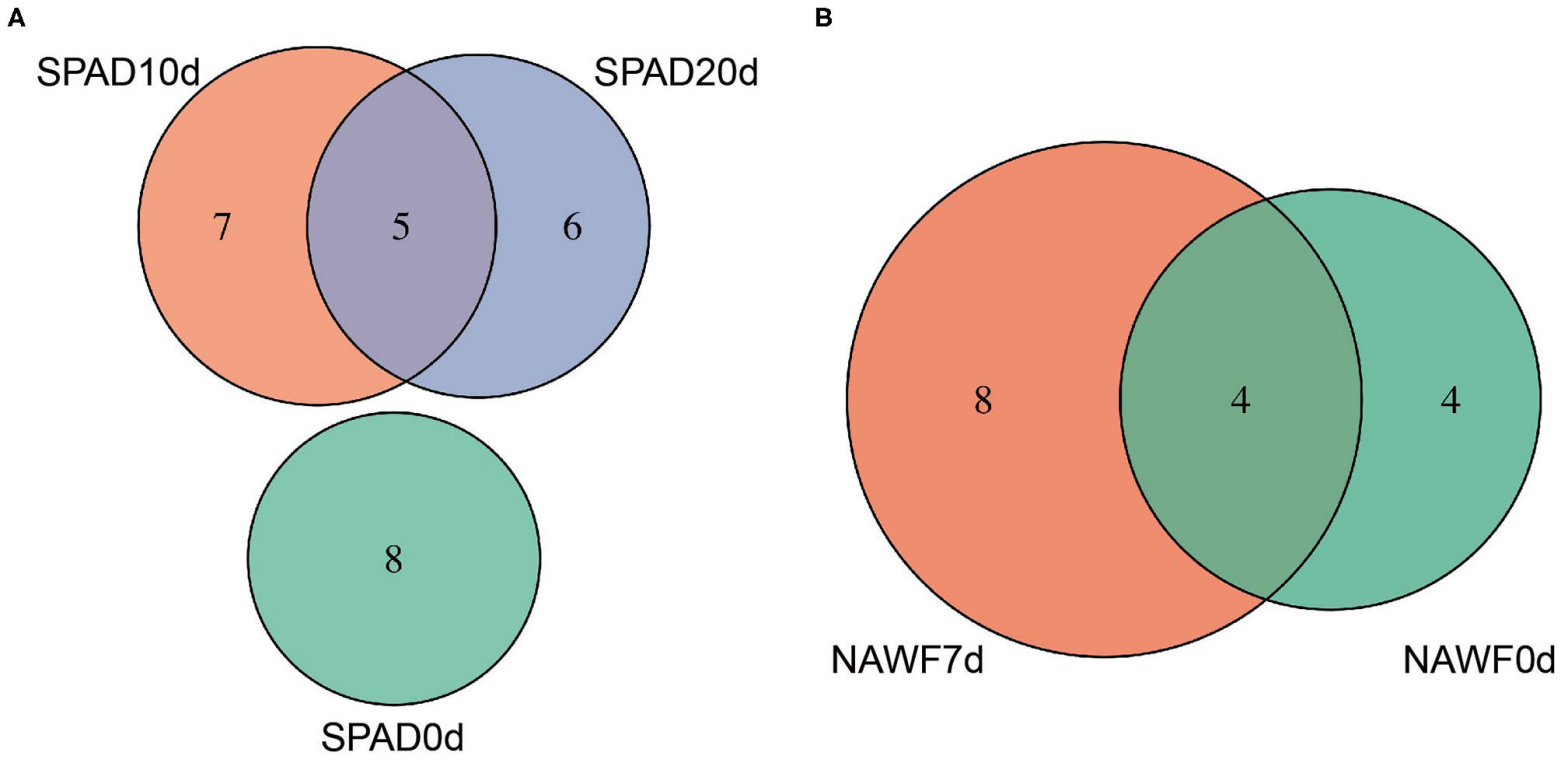
Venn diagram of genomic loci for **(A)** SPAD and **(B)** NAWF traits.

All GWAS risk loci were located on 22 chromosomes, spanning a total of ~51.50 megabases (Mb). For each chromosome, there were 1~4 loci, except chromosome A12, A13, D09, and D11, and chromosome A08 and D08 contained the highest number of genomic loci (4), spanning 2.95 and 5.10 Mb, respectively (Supplementary Table S5). Although upland cotton is an allotetraploid, we did not observe a significant difference in the number of GWAS loci between the At and Dt subgenomes, and each of these subgenomes harbored 25 genomic loci. As expected, we observed some genomic regions were associated with multiple traits, such as A02_105891088_107196428 (associated with C2013SPAD10d, C2014SPAD20d, X2014RSPAD, SPAD10d_blup and C2013NAWF0d) and D03_37952328_38393621 (associated with C2013NAWF0d, NAWF0d_blup, NAWF7d_blup, NB_blup and RSPAD_blup). Interestingly, the locus D13_59408561_60730103 was associated with three senescence traits (C2013NAWF7d, NAWF0d_blup and NAWF7d_blup) and two production traits (X2014MBW and C2014MBW), which indicates the possibility of this locus containing different genes controlling senescence and production traits, or a single gene with pleiotropic effect. In addition, distinct associations were observed at different time points. For example, there were 8, 12, and 11 loci associated with SPAD values at 0, 10, and 20 days after topping, respectively. Interestingly, five loci were shared between SPAD10d and SPAD20d but there was no overlap with SPAD0d (Figure 3A), suggesting that significant changes occurred during growth and development of cotton plants. Similarly, NAWF values were associated with more genomic loci on the 7th after topping than on the topping day, although they shared four genomic loci (Figure 3B).

### 3.3. Candidate Gene Prediction

In this study, genes located in 50 genomic risk loci were selected for the identification of senescence candidates, and a total of 2120 genes were identified. We then performed a GO enrichment analysis of all the selected genes (Supplementary Figure S7). The top three significant GO terms were GO:1901698 (response to nitrogen compound), GO:0048583 (regulation of response to stimulus), and GO:0042742 (defense response to bacterium). These results show that the process of cotton senescence is a complex multichannel process. We also found that 409 genes were recorded in Leaf Senescence Database LSD 3.0 (Li et al., 2020) using the homologous genes of Arabidopsis (Supplementary Table S6), which included reported senescence-associated genes such as *SAG12, SAG20* and *SAG101*. Moreover, members of transcription factor families that regulate the process of plant senescence were identified such as *WRKY75* (Guo et al., 2017), *bHLH105* (Aparicio and Pallás, 2017), *MYB15* (Medina et al., 2011), and *NAC029* (Kim et al., 2009). These results demonstrated the effectiveness of our approach to dissecting the genetic basis of cotton senescence.

**Figure 4:**
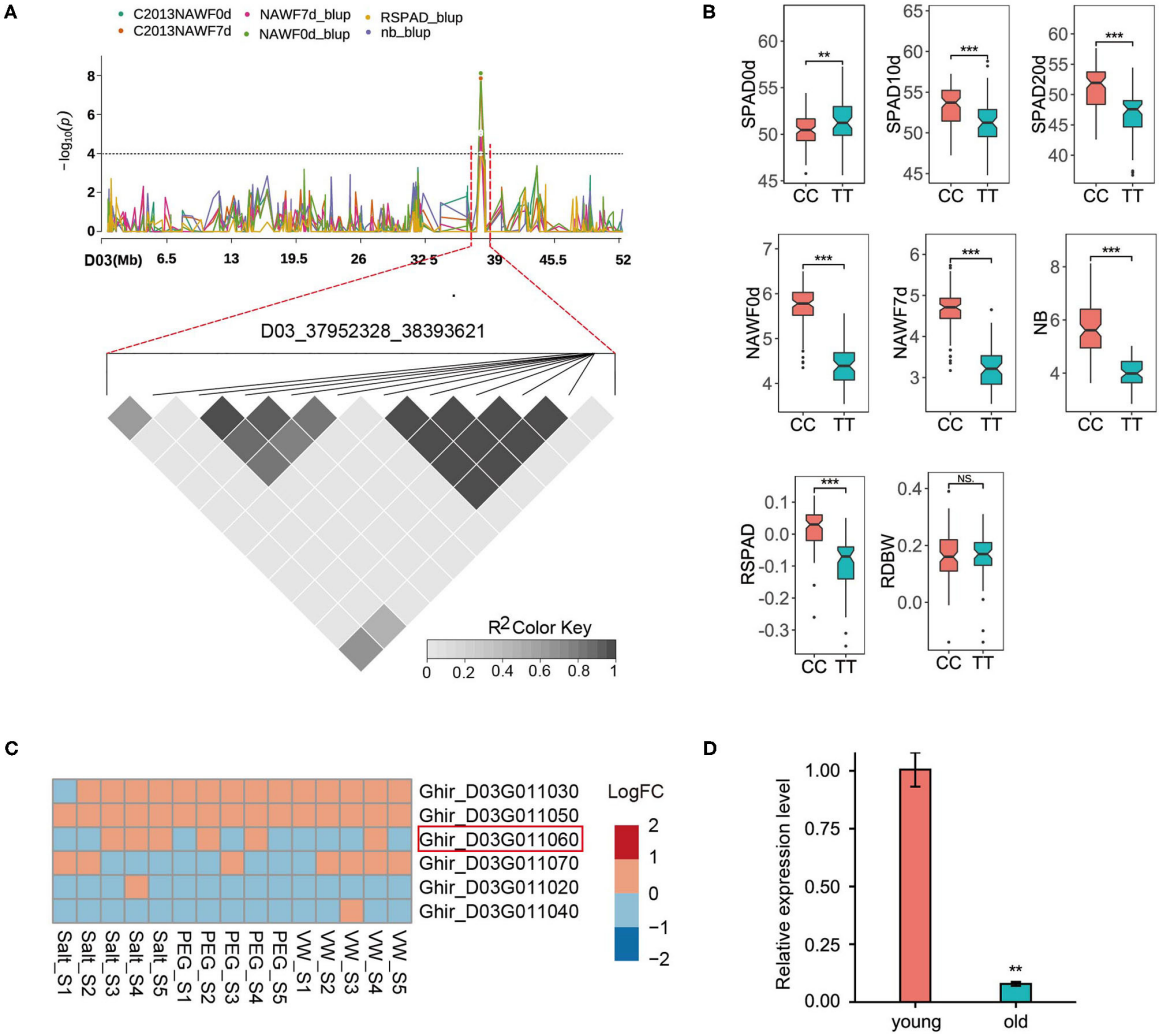
GWAS for senescence-related traits and identification of the candidate genes on chromosome D03. **(A)** Manhattan plot of multi-traits (top) and LD heat map (bottom) of the genomic risk locus D03_37952328_38393621. **(B)** Box plots for eight senescence-related traits based on the genotypes of the SNP Ghir_D03_37952328. 61 accessions for the TT allele and 93 accessions for the CC allele. **(C)** Heatmap of the expression patterns of six predicted genes using the RNA-seq data. Red indicates up-regulated expression, and blue indicates down-regulated expression. S1 - S5: 6, 12, 24, 48, and 72 h after the corresponding treatment. **(D)** Expression of Ghir_D03G011060 in young and old leaves by qRT-PCR. Asterisks indicate significance levels (***P < 0.001, **P < 0.01, and *P < 0.05), NS, not significant.

D03_37952328_38393621 represented a highly promising locus, as it could be detected in multiple senescence traits (Figure 4A). The peak SNP (Ghir_D03_37952328) produced two homozygous alleles, CC and TT. Compared with the accessions (61) carrying the TT allele, the other accessions (93) carrying the CC allele exhibited a significant increase in phenotype values in most traits, however, a significant decrease in SPAD0d was observed (Figure 4B). This locus contained nine predicted genes, and six genes were expressed (*logCPM*>1) in the RNA-seq data (Figure 4C). Although these genes showed small changes between the control group and the different treatment groups, *Ghir_D03G011060* was the most likely candidate gene based on gene function. Ghir_D03G011060 is homologous to AT5G23040 in Arabidopsis and encodes a cell growth defect factor-like protein (CDF1). AT5G23040 was annotated as a cell death process (GO: 0008219), and has been reported to play an important role in chlorophyll synthesis (Lee et al., 2013; Reinbothe et al., 2015). Among the chlorophyll biosynthetic pathways in higher plants, the only light-dependent step is the reduction of protochlorophyllide (Pchlide) to chlorophyllide (Chlide) catalyzed by the NADPH: protochlorophyllide oxidoreductase (POR) (Reinbothe et al., 2010). The CDF1 protein is identified as a chaperone that assists the translocation of the PORA protein, one of the three POR isoforms (the other two are PORB and PORC), and it regulates the response of Pchlide homeostasis to lower the photoexcitative damage (Reinbothe et al., 2015). To further investigate the expression pattern of candidate genes, qRT-PCR was performed to detect the transcript levels of Ghir_D03G011060 in the various development stages of the leaves. The results showed that the expression level of Ghir_D03G011060 in old leaves was significantly lower than that in young leaves (Figure 4D), suggesting that Ghir_D03G011060 is involved in the process of leaf senescence in cotton.

The other two notable genomic loci were A02_105891088_107196428 and D13_59408561_60730103. First, within the locus A02_105891088_107196428 (Supplementary Figure S8A), the peak SNP, Ghir_A02_106204996, had two homozygous alleles, CC (38 accessions) and TT (118 accessions). Significant phenotypic differences were observed between the accessions carrying the CC and TT alleles for multiple traits (Supplementary Figure S8B). The TT allele showed higher NAWF, NB and SPAD values. Although there were 104 genes located on the locus A02_105891088_107196428, 60 genes were expressed (*logCPM*>1) with varying degrees of up-regulation or down-regulation in the RNA-seq data after stress treatments (Supplementary Figure S8C). Among these, Ghir_A02G017660 was the most likely candidate gene for the locus A02_105891088_107196428, as it was significantly upregulated (*logFC*>1) under all stress treatments. Ghir_A02G017660 is homologous to Arabidopsis AT2G23450 (*WAKL14*), which encodes a protein kinase. Function prediction showed that Ghir_A02G017660 was involved in the protein phosphorylation process (GO:0006468). Moreover, Ghir_A02G017660 was recorded as a senescence-associated gene in the LSD 3.0 database.The locus D13_59408561_60730103 was only significantly associated with NAWF and MBW (Supplementary Figure S9A). Nevertheless, two homologous alleles (AA and GG) of the peak SNP (Ghir_D13_60292895) showed significant phenotypic differences in NAWF and MBW as well as NB and RSPAD (Supplementary Figure S9B). In this genomic region, there were 74 expressed genes (*logCPM*>1) that showed distinct expression patterns under different treatments (Supplementary Figure S9C). Among them, 12 genes were found in the LSD 3.0 database using their homologous genes in Arabidopsis (Supplementary Table S6), and four genes (Ghir_D13G021720, D13G022030, D13G022290 and D13G022430) showed different expressions (|*logFC*>1|) in the RNA-seq data (Supplementary Figure S9C). Interestingly, we observed a nonsynonymous SNP (D13_59468165) in the exon of the gene Ghir_D13G021720, which caused a change from A to G as well as from arginine (AGA) to glycine (GGA). This variation was also found in the Cotton Omics Database (http://cotton.zju.edu.cn/index.htm) (named v-gh-D13-60405265 with 0.15 allele frequency). Ghir_D13G021720 is a homolog of the Arabidopsis abscisic acid-insensitive2 (*ABI2*), which encodes a protein phosphatase 2C and is involved in ABA signal transduction. A recent report revealed that *ABI2* is involved in the inhibition of brassinosteroid (BR) signaling by ABA in which ABI2 interacts with dephosphorylate BIN2, a negative regulator of BR signaling (Wang et al., 2018). Another study reported that transmembrane kinase protein 4 (TMK4) could negatively regulate the ABA signaling pathway by phosphorylating ABI2 at three conserved Ser residues (Li et al., 2021a). Overall, we considered Ghir_D13G021720 as the most likely candidate gene for locus D13_59408561_60730103.

### 3.4. Potential Functional Roles of GhCDF1 in Cotton Senescence

**Figure 5:**
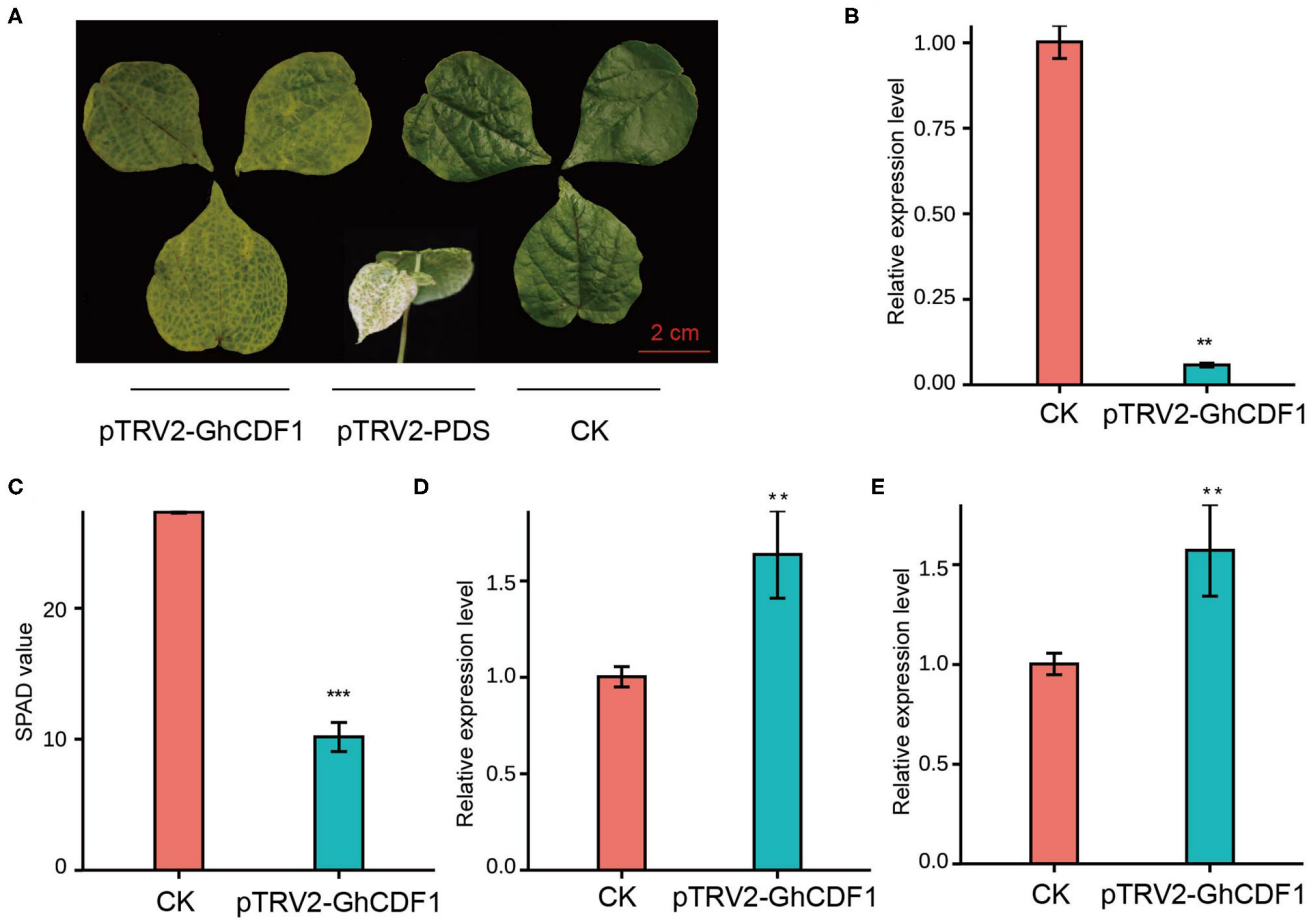
Functional analysis of the candidate gene *GhCDF1* (Ghir_D03G011060) using VIGS. **(A)** Plant phenotype of VIGS *GhCDF1* in cultivar 'CRI50'. 'CRI50' with pTRV2 was used as the control (CK). **(B)** Expression of *GhCDF1* in VIGS plants. **(C)** SPAD values of the VIGS plants. **(D)** and **(E)** Expression of senescence-associated genes *GhWRKY27* and *GhWRKY42*. Asterisks indicate significance levels (***P < 0.001, **P < 0.01, and *P < 0.05).

To further investigate the function of Ghir_D03G011060 (*GhCDF1*) in cotton senescence, we constructed the recombinant virus pTRV2-GhCDF1 and performed virus-induced gene silencing (VIGS). As an indicator, plants infected with pTRV2-PDS exhibited a bleaching phenotype, indicating that our system was effective (Figure 5A). Compared with CK plants, the gene-silenced plants (pTRV2-GhCDF1) showed a significantly lower expression level of Ghir_D03G011060 (Figure 5B), as well as highly evident leaf chlorosis and a significant decrease in the SPAD value (Figures 5A,C). In addition, the expression levels of the two senescence-associated genes (*GhWRKY27* and *GhWRKY42*) were detected, which are upregulated during leaf senescence in cotton (Gu et al., 2018, 2019a). In our study, *GhWRKY27* and *GhWRKY42* markedly increased in the silenced plants (Figures 5D,E), indicating that silencing of the endogenous Ghir_D03G011060 gene promoted leaf senescence in cotton.

## 4. Discussion

During the past decade, genome-wide association studies have become a highly effective technique for the genetic dissection of complex quantitative traits in crop species and have become a powerful and ubiquitous technique (Tibbs Cortes et al., 2021). As an important natural fiber crop, the genetic basis of many agronomic traits in cotton has been revealed by GWAS, such as fiber yield, fiber quality, flowering time, plant architecture and stress tolerance (Fang et al., 2017; Li et al., 2017, 2021b; Ma et al., 2018; Su et al., 2018; Abdelraheem et al., 2021). Nevertheless, there is a lack of understanding of the genetic architecture and genomic regions involved in cotton senescence.

In this study, a genome-wide association study of cotton senescence was performed for the first time. Given the complexity of the senescence mechanism (Jansson and Thomas, 2008), we investigated eight traits/indices for estimating cotton senescence in multiple environments, including SPAD0d, SPAD10d, SPAD20d, RSPAD, NAWF0d, NAWF7d, NB, and RDBW. The chlorophyll content always reached a maximum during leaf growth and development, and then decreased until the leaves turned yellow. Therefore, SPAD, a relative chlorophyll content value, is typically used to evaluate the degree of leaf senescence (Hebbar et al., 2005; Mart́ınez et al., 2008; Zhao et al., 2019). NAWF reflects the growth and development of cotton plants; therefore, it can also be used as an indicator of cotton senescence (Dong et al., 2005; Thompson et al., 2019). Compared with normal senescence cotton, premature senescence cotton always shows a smaller number of upper bolls and a larger difference in boll weight between the upper and middle parts (Dong et al., 2005), and therefore NB and RDBW were investigated. In this study, we detected the lowest SPAD value (49.49±5.57) and maximum coefficient of phenotypic variation (11%) on the 20th day after topping (Supplementary Table S2). Therefore, the SPAD value obtained for this period could be used as a reference for cotton senescence. In addition, NAWF, NB and RSPAD were highly correlated with SPAD20 (*r*>0.5, *P* < 0.001) (Figure 2), suggesting that these indices could also be used to diagnose senescence in cotton.

Considering that the advantages of multi-locus models for complex traits (Wen et al., 2018; Zhang et al., 2019), we performed a multi-locus GWAS analysis to identify QTNs associated with cotton senescence using the FASTmrMLM method. A total of 63 significant and stable QTNs were detected (Supplementary Table S4), which represented 50 genomic regions located on 22 chromosomes except A12, A13, D09, and D11 (Supplementary Table S5). This result showed that the progress of cotton senescence is controlled by a highly complex regulatory mechanism. Interestingly, we observed that several genomic regions were co-localized with senescence traits and yield traits (UBW and MBW). For example, the locus A03_433214_663224 was associated with X2014RSPAD, C2014UBW, NAWF0d_blup, and X2014NAWF7d (Supplementary Table S5). These results suggest that senescence and production in cotton are partially regulated by common genetic mechanisms, although the data of yield traits come from only two environment. More importantly, we detected three steady and reliable genomic loci (D03_37952328_38393621, A02_105891088_107196428, and D13_59408561_60730103), and then three highly likely candidate genes were then obtained.

In the genomic region D03_37952328_38393621, the Ghir_D03G011060 encodes a cell growth defect factor-like protein (CDF1) and is a homologous gene of AT5G23040 in *Arabidopsis thaliana*. *CDF1* was first identified as a possible cell death inducer by screening the Arabidopsis cDNA in yeast, and was observed to induce ROS generation and cause yeast cell death (Kawai-Yamada et al., 2005). *CDF1* is essential in Arabidopsis embryogenesis because of its embryo lethality in mutants (Kawai-Yamada et al., 2014). In addition, *CDF1* deficiency by DEX-inducible RNAi results in a decrease in the PORA protein and defective chlorophyll synthesis under light conditions (Lee et al., 2013; Reinbothe et al., 2015). In this study, *CDF1* was chosen as the candidate gene affecting cotton senescence, and has also been observed in a genome-wide association study of salt tolerance in Autotetraploid Alfalfa (Liu et al., 2019) and QTL mapping of flowering dates in barley (Monteagudo et al., 2019). Additionally, *CDF1* is downregulated under cold acclimation using mass spectrometry-based proteomics (Trentmann et al., 2020). In our study, Ghir_D03G011060 was dramatically downregulated in old cotton leaves by qRT-PCR (Figure 4D), and the Ghir_D03G011060-silenced plants (pTRV2-GhCDF1) showed the loss of green leaves as well as lower SPAD values and upregulated expression of senescence marker genes (*GhWRKY27* and *GhWRKY42*) (Figure 5). Together, these results highlight the important role of Ghir_D03G011060 in cell death and the regulation of the cotton senescence process. Furthermore, two candidate genes, Ghir_A02G017660 and Ghir_D13G021720, were identified in A02_105891088_107196428 and D13_59408561_60730103, respectively. Ghir_A02G017660 is homologous to AT2G23450 in *Arabidopsis thaliana*. This gene belongs to the wall-associated kinase (WAK) gene family and encodes a WAK-like protein named WAKL14. Members of WAKs exhibit a wide range of functions in plants, including cell expansion, pathogen response, and disease response (Meier et al., 2010; Kohorn and Kohorn, 2012; Rosli et al., 2013; Zuo et al., 2015). In rice, *OsWAK14* has been reported to be mediated by the fungal chitin and positively regulates the quantitative resistance of the rice blast fungus (Delteil et al., 2016). In our study, the expression level of *GhWAKL14* (Ghir_A02G017660) increased to varying degree under different stresses by RNA-seq, particularly in cases of PEG treatment and inoculation with *Verticillium Wilt* (Supplementary Figure S8C), but no significant difference was found between young and old leaves by qRT-PCR (Supplementary Figure S10A). One possible for the disaccord is that stress signals are required for the high-level expression of *GhWAKL14*. These results suggest that Ghir_A02G017660 may be involved in stress-induced leaf senescence, especially biotic stress, however, further studies are needed to better understand its regulatory mechanism in the senescence process. Ghir_D13G021720 encodes a protein phosphatase 2C family protein (PP2C) and is a homolog of *ABI2* in Arabidopsis. PP2Cs are involve in the abscisic acid (ABA) signaling pathway as negative regulators (Li et al., 2012; Cai et al., 2014) and suppress stress signaling under unstressed or mild stress conditions (Fujii and Zhu, 2009; Rubio et al., 2009; Umezawa et al., 2009; Li et al., 2021a). In our study, we found that *GhABI2* (Ghir_D13G021720) was significantly downregulated in the RNA-seq data under all stress treatments (PEG, salt and VW), suggesting that it may have a similar function to its homologous gene in Arabidopsis. Moreover, the expression level of Ghir_D13G021720 in old leaves was also significantly downreuglated than that in young leaves by qRT-PCR (Supplementary Figure S10B). Interestingly, a nonsynonymous SNP (D13_59468165) was found in the CDS region of Ghir_D13G021720 and was confirmed by independent research in the Cotton Omics Database. Therefore, the function of Ghir_D13G021720 and nonsynonymous variation in cotton senescence is worthy of further study.

## Data Availability Statement

The datasets presented in this study can be found in online repositories. The names of the repository/repositories and accession number(s) can be found in the article/Supplementary Material.

## Author Contributions

SY, ZF, and QL designed the project and revised the manuscript. QL and LL performed the experiments. QL performed data analysis and wrote the manuscript. All authors contributed to the article and approved the submitted version.

## Conflict of Interest

The authors declare that the research was conducted in the absence of any commercial or financial relationships that could be construed as a potential conflict of interest.

## Publisher's Note

All claims expressed in this article are solely those of the authors and do not necessarily represent those of their affiliated organizations, or those of the publisher, the editors and the reviewers. Any product that may be evaluated in this article, or claim that may be made by its manufacturer, is not guaranteed or endorsed by the publisher.
