# Supplementary material for: Uncovering Novel Genomic Regions and Candidate Genes for Senescence-Related Traits by Genome-Wide Association Studies in Upland Cotton (Gossypium hirsutum L.)
Source: Front Plant Sci. 2022 Jan 5;12:809522. doi: 10.3389/fpls.2021.809522 (PMC8766411; doi:10.3389/fpls.2021.809522)
Supplement: Supplementary file 2 [file Data_Sheet_1.PDF]

Supplementary Material

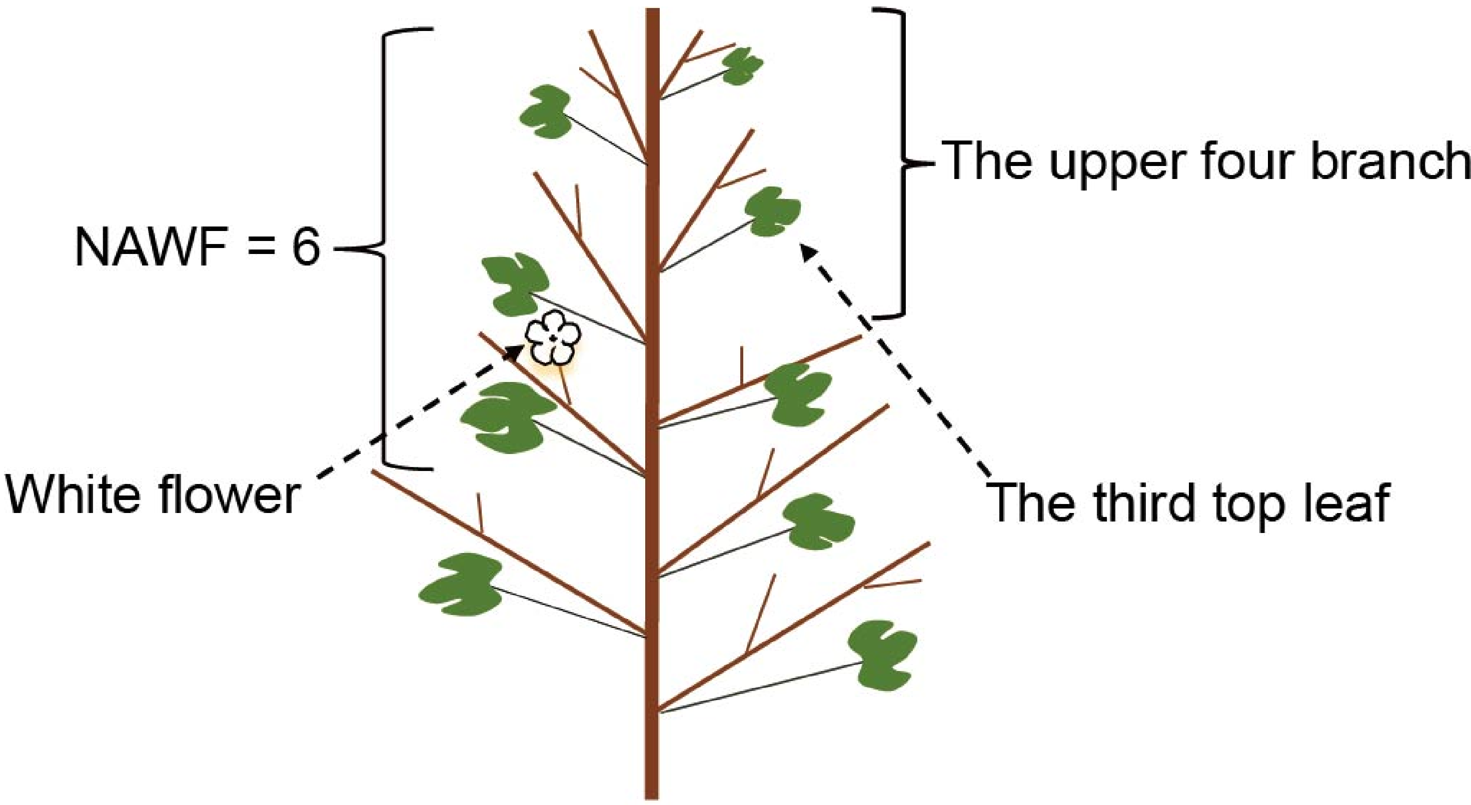

Figure S1. A cotton sketch for the traits investigated. SPAD values of the third top leaf were measured. The number of open bolls on the upper four branches was counted. when the white flower is in the position shown, the number of nodes above white flower (NAWF) is 6.

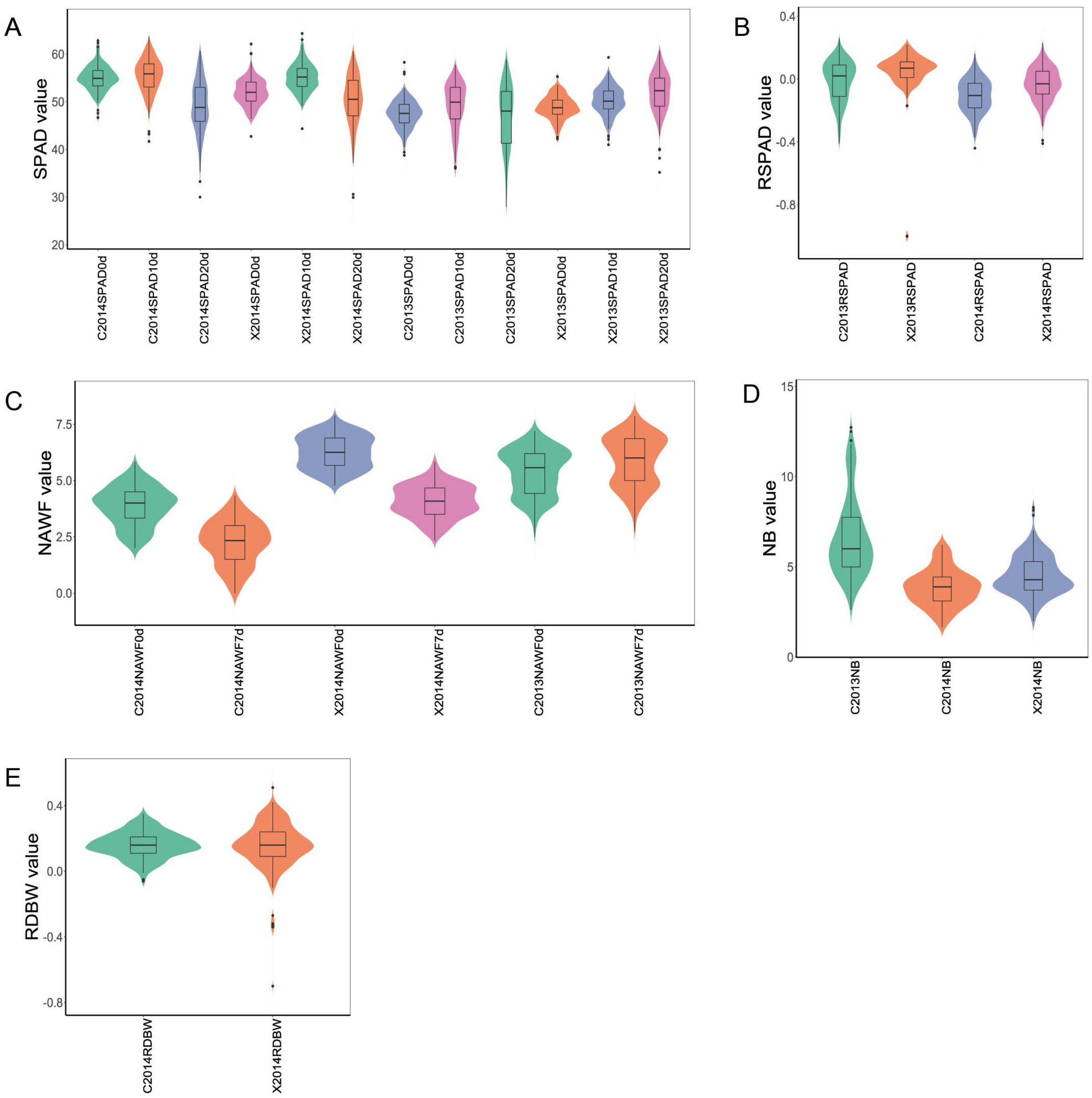

Figure S2. Phenotypic distributions of all senescence-related traits among 185 cotton accessions. **(A)** Distributions of SPAD values at three stages (0, 10, 20 days after topping). **(B)** Distributions of the relative SPAD difference (RSPAD) value. **(C)** Distributions of nodes above white flower (NAWF) value at two stages (0 and 7 days after topping). **(D)** Distributions of the number of open bolls on the upper four branches (NB) value. **(E)** Distributions of relative difference of boll weight (RDBW) value.

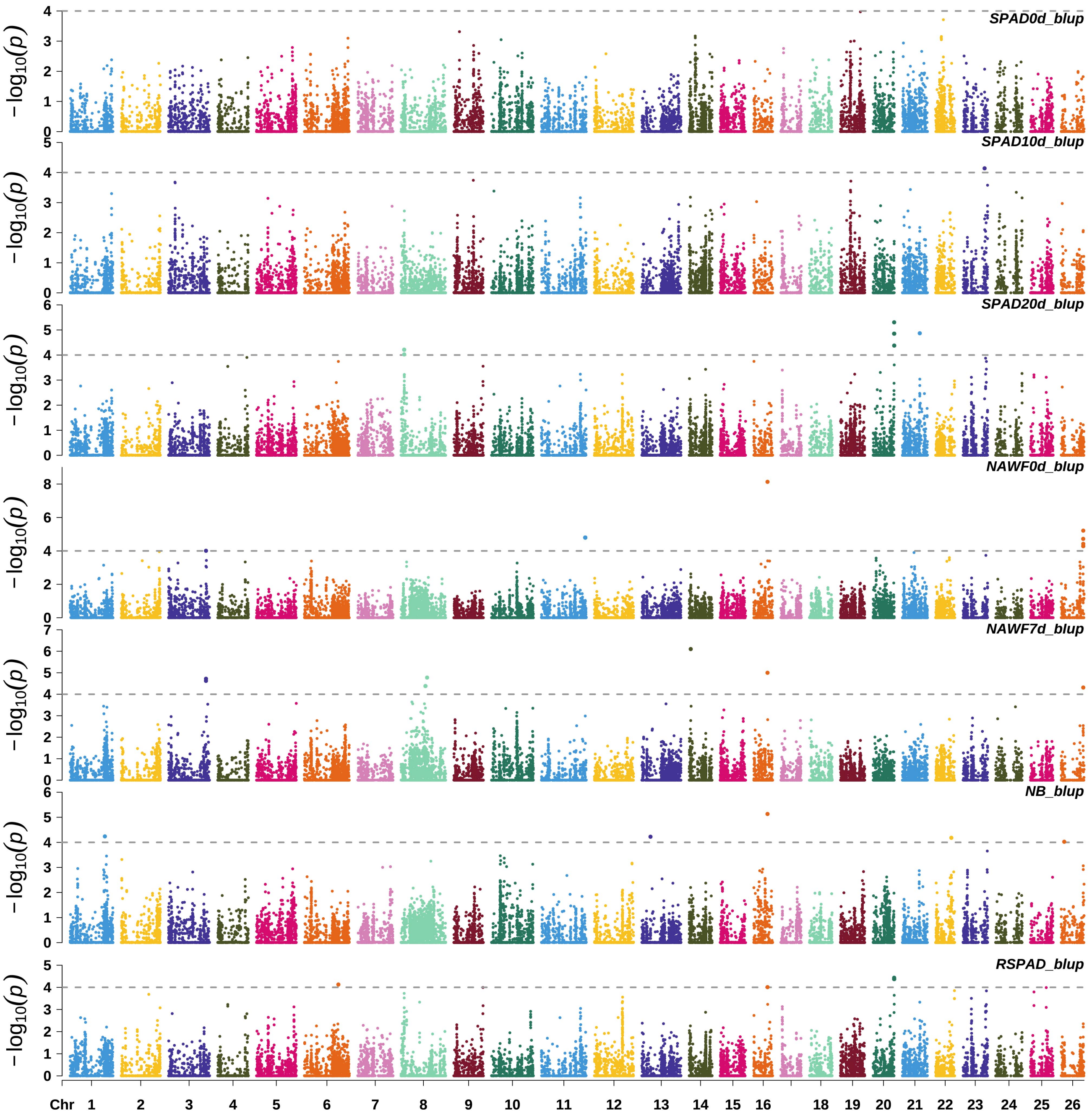

Figure S3. Manhattan plots for BLUP of senescence-related traits. Nodes above white flower at 0 day and 7 days after topping (NAWF0d and NAWF7d), number of open bolls on the upper four branches (NB), relative SPAD difference (RSPAD), SPAD value at 0, 10 and 20 days after topping (SPAD0d, SPAD10d and SPAD20d).

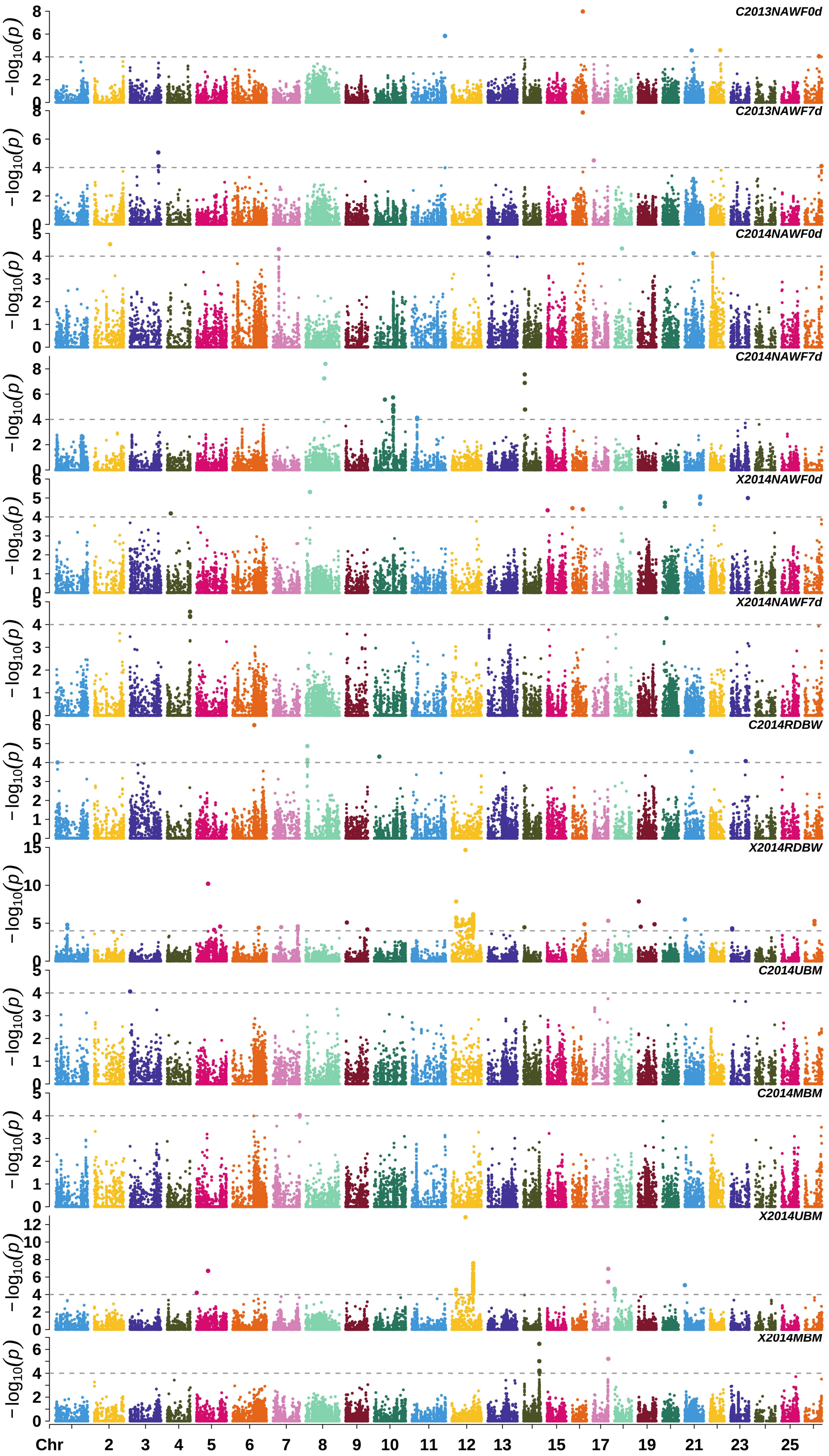

Figure S4. Manhattan plots for single environmental phenotype of senescence-related traits. Relative difference of boll weight (RDBW), upper boll weight (UBW), middle boll weight (MBW).

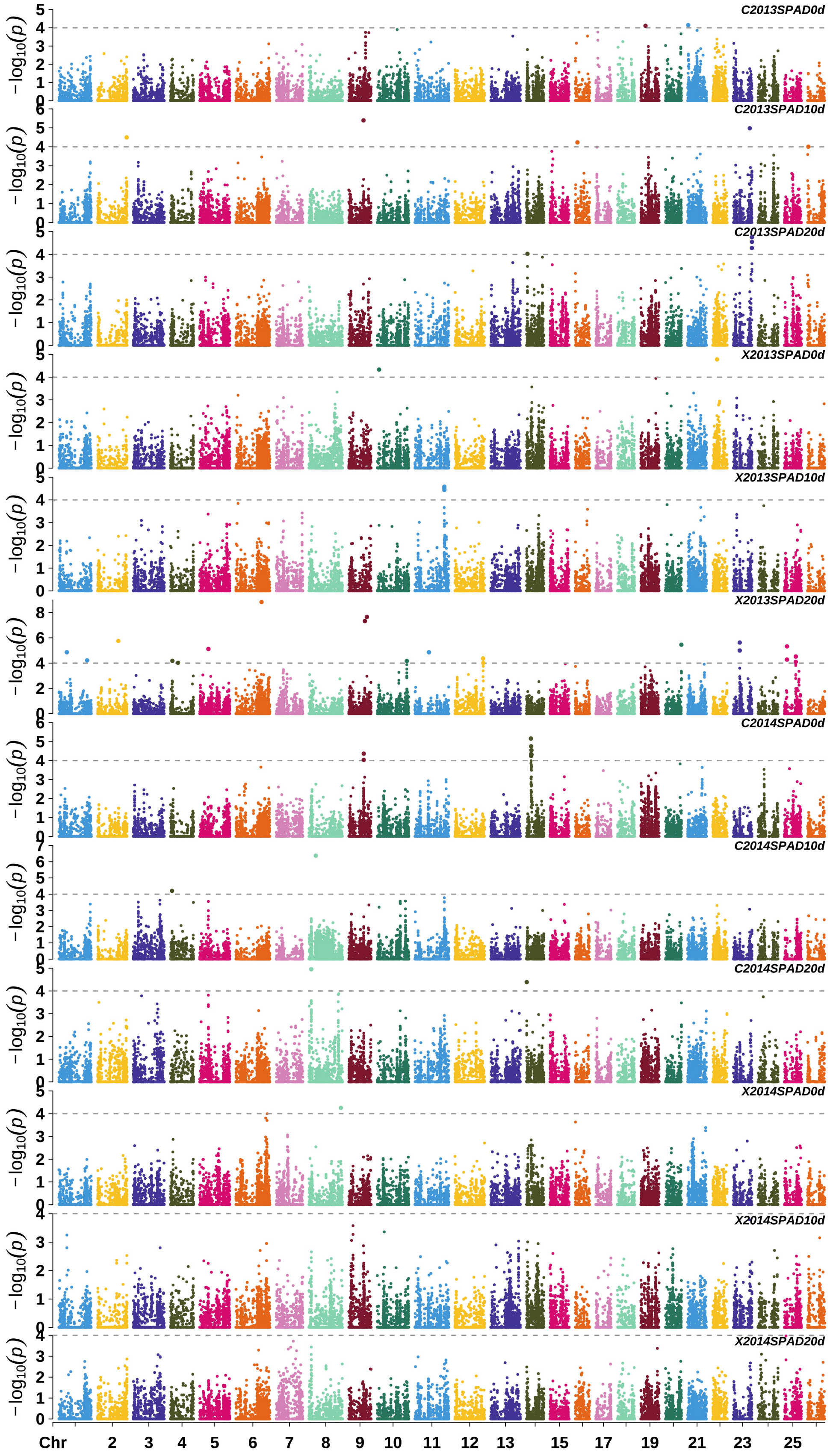

Figure S5. Manhattan plots for single environmental phenotype of senescence-related traits

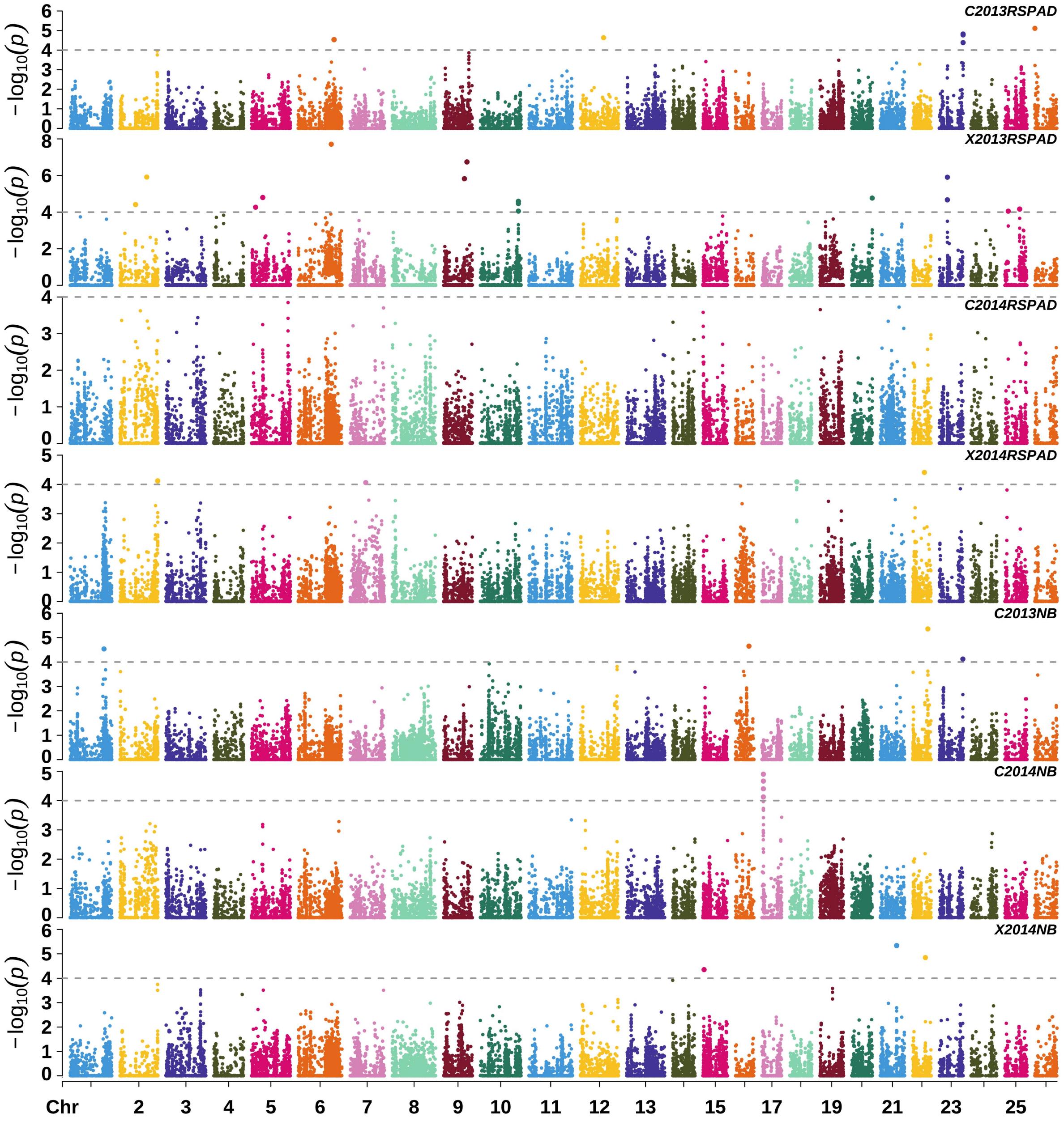

Figure S6. Manhattan plots for single environmental phenotype of senescence-related traits.

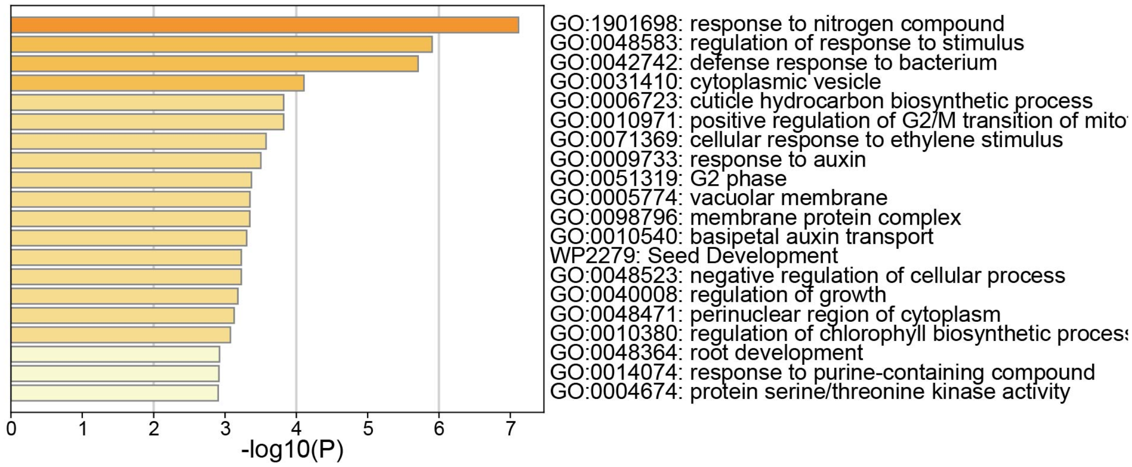

Figure S7. GO enrichment analysis of all candidate genes.

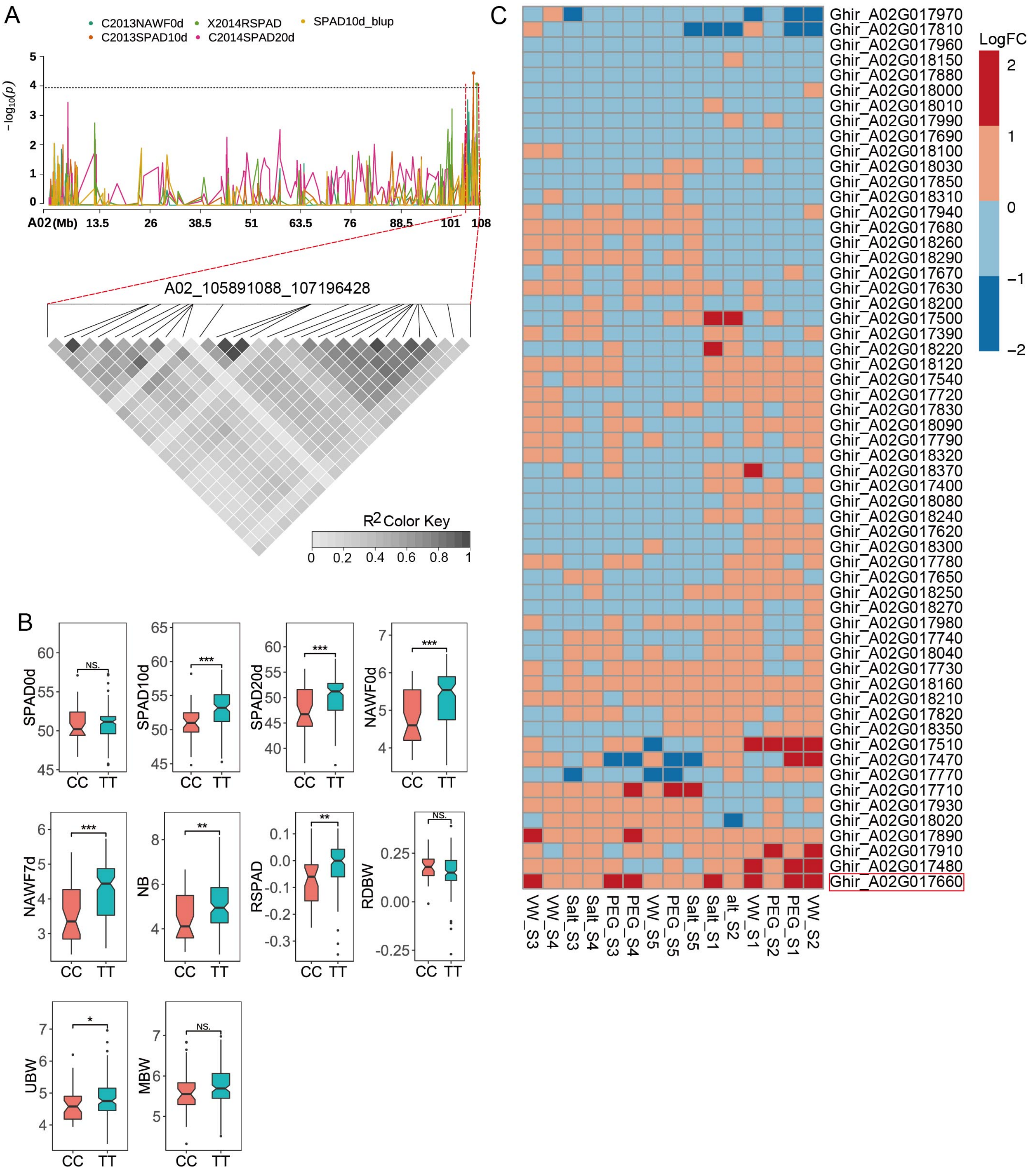

Figure S8. GWAS for senescence-related traits and identification of the candidate genes on chromosome A02. **(A)** Manhattan plot of multi-traits (top) and LD heat map (bottom) of the genomic risk locus A02_105891088_107196428. **(B)** Box plots for eight senescence-related traits and two yield-related traits (UBW and MBW), based on the genotypes of the SNP, A02_106204996. 38 accessions for the CC allele and 118 accessions for the TT allele. UBW: upper boll weight, MBW: middle boll weight. **(C)** Heatmap of expression patterns of six predicted genes using the RNA-seq data. Red indicates upregulated expression, and blue indicates downregulated expression. S1 - S5: 6, 12, 24, 48 and 72 h after the corresponding treatment. *** $P < 0.001$, ** $P < 0.01$, * $P < 0.05$, NS: not significant.

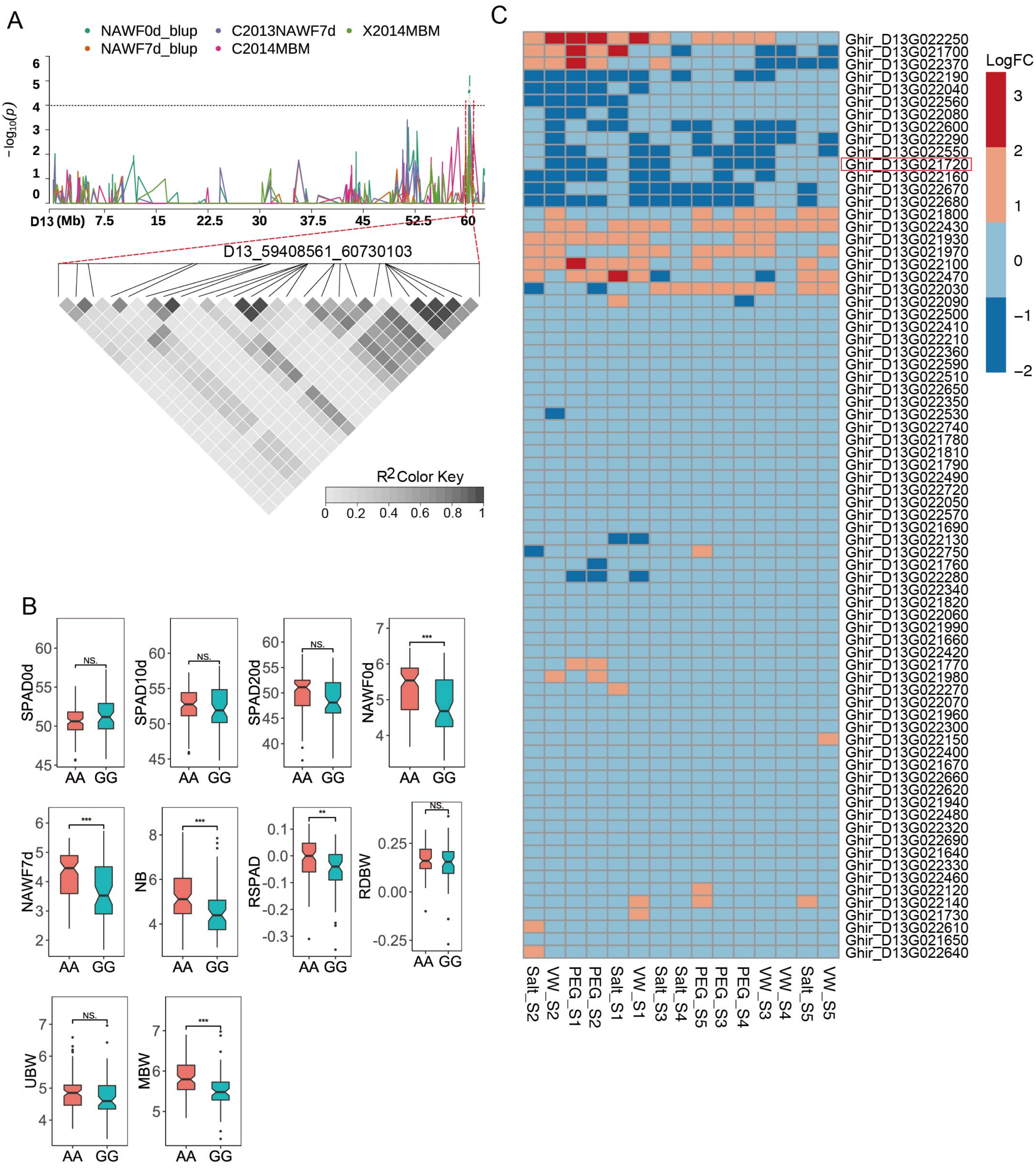

Figure S9. GWAS for senescence-related traits and identification of the candidate genes on chromosome D13. **(A)** Manhattan plot of multi-traits (top) and LD heat map (bottom) of the genomic risk locus D13_59408561_60730103. **(B)** Box plots for eight senescence-related traits and two yield-related traits (UBW and MBW), based on the genotypes of the SNP, Ghir_D13_60292895. 106 accessions for the AA allele and 62 accessions for the GG allele. UBW: upper boll weight, MBW: middle boll weight. **(C)** Heatmap of expression patterns of six predicted genes using the RNA-seq data. Red indicates upregulated expression, and blue indicates downregulated expression. S1 - S5: 6, 12, 24, 48 and 72 h after the corresponding treatment. *** $P < 0.001$, ** $P < 0.01$, * $P < 0.05$, NS: not significant.

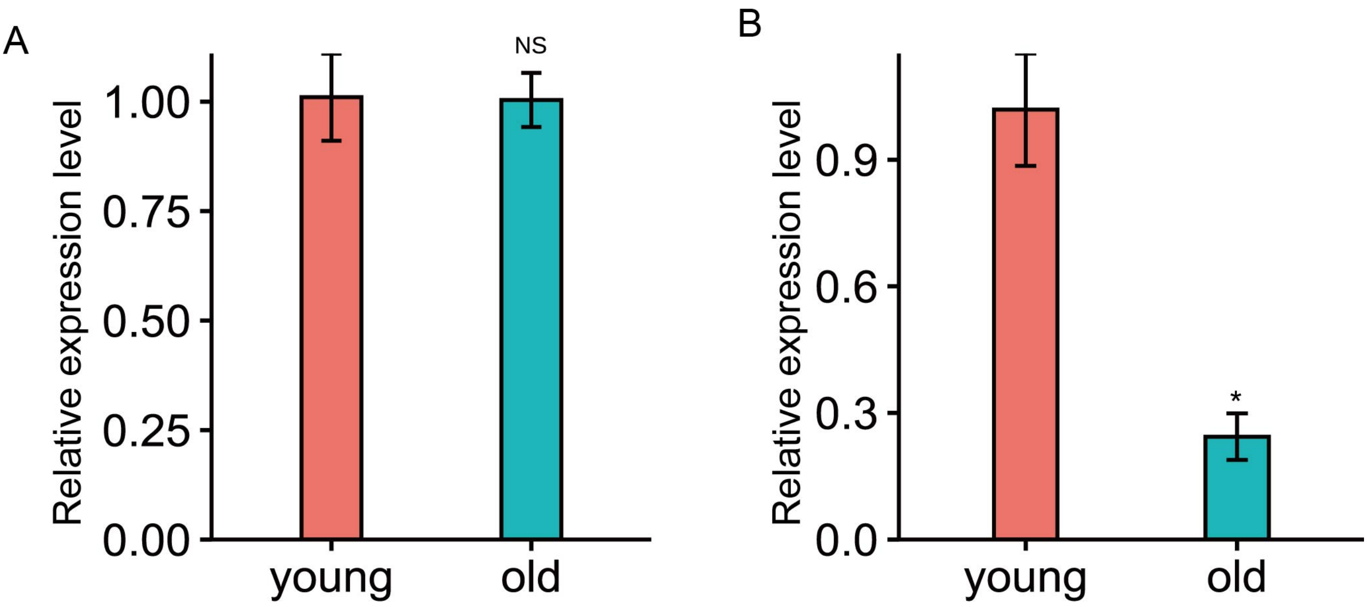

Figure S10. Expression of Ghir_A02G017660 (**A**) and Ghir_D13G021720 (**B**) in young and old leaves by qRT-PCR. * $P < 0.05$, NS: not significant.

Figure S11. Enter the caption for your figure here. Repeat as necessary for each of your figures
